# Prevalence and risk factors of *Cryptosporidium* spp. and *Giardia* infestation in cattle and in broiler chickens in Algeria

**DOI:** 10.17221/82/2024-VETMED

**Published:** 2025-02-24

**Authors:** Ratiba Baazizi, Messaouda Taibi, Nour Senouci, Djamel Baroudi, Sarah Khodja, Redha Belala, Djamel Khelef, Nora Mimoune

**Affiliations:** ^1^HASAQ Laboratory, Higher National Veterinary School, Algiers, Algeria; ^2^Animal Health and Production Laboratory (SPA), Higher National Veterinary School, Algiers, Algeria; ^3^Biotechnologies Platform for Animal Medicine and Reproduction (BIOMERA), Saad Dahleb Blida University, Blida, Algeria; ^4^Biotechnologies Laboratory Related to Animal Reproduction (LBRA), Institute of Veterinary Sciences, Saad Dahleb, Blida University, Blida, Algeria

**Keywords:** broiler chickens, calves, giardiasis, prevalence, risk factors cryptosporidiosis

## Abstract

Cryptosporidiosis and giardiasis are zoonotic protozoan diseases with significant public health and economic concerns. In Algeria, epidemiological data on these parasites in livestock are limited. This study aimed to estimate the prevalence of *Cryptosporidium* spp. and *Giardia* in dairy calves and broiler chickens and to identify the associated risk factors. A microscopic analysis of 200 faecal samples revealed a *Cryptosporidium* spp. prevalence of 56% in calves and 60% in broiler chickens, while the *Giardia* prevalence was 8% in calves and absent in chickens. In cattle, the data showed that age constituted a potential risk factor for both parasites (*P* < 0.000 1 for *Cryptosporidium*; *P* < 0.000 5 for *Giardia*). Interestingly, the risk of a *Cryptosporidium* infection decreased with age, while the *Giardia* infestation risk increased. The sex was not a significant factor for *Cryptosporidium* (*P* = 0.115 0), but was impactful for *Giardia* (*P* < 0.000 1), with males at higher risk. These results highlighted the distinct epidemiological characteristics of *Cryptosporidium* and *Giardia* infestations in Algerian livestock. The contrasting age-related risks and sex-specific susceptibility to *Giardia* underline the need for targeted, age and sex prevention strategies. This study provides valuable data to inform public health policies and to improve livestock management practices in Algeria, contributing to the wider understanding of these zoonotic parasites in North African agricultural farming.

*Cryptosporidium* spp*.* and *Giardia duodenalis* can cause asymptomatic infections or mild to severe gastrointestinal disorders in humans and animals. These two parasites are of concern for animal health due to the direct economic losses. They also pose a public health problem due to the risk of human exposure to environmental contamination by zoonotic species. For *Cryptosporidium*, of the 44 valid species and 120 genotypes, 19 species and four genotypes have been reported in humans with the most prevalent being: *C.* *parvum, C.* *hominis, C. meleagridis, C.* *canis* and *C.* *felis (*Ryan et al. 2021). For *Giardia* sp*.*, *Giardia duodenalis* is the species which affects mammals and is the most known zoonotic for birds and mammals (Feng and Xiao 2011; Egan et al. 2024).

In addition to diarrhoea, the most commonly associated symptoms of giardiasis and cryptosporidiosis are loss of appetite, fever, and malabsorption. These infections can impact the weight gain of the affected animals (Santin 2020) and can lead to mortality, resulting in economic losses. Cryptosporidiosis is also responsible for economic losses in the poultry industry, where episodes of morbidity and even mortality in industrial birds are regularly reported (Guechtouli et al. 2022). The transmission of these diseases, whether *Cryptosporidium* spp. or *Giardia*, occurs through the oral-faecal route, either by direct contact with an infected individual or by consuming water or food contaminated with oocysts or cysts (Santin 2020). The growing interest in the study of these parasites in livestock animals is no longer solely motivated by the knowledge of their zoonotic potential, but by the need to evaluate the risk factors associated with parasitosis in animals to implement appropriate hygiene and preventive measures and to reduce their impact in livestock production (Bennadji et al. 2022).

Several studies have been conducted on *Cryp-tosporidium* in chickens. Three species are recognised: the first, *Cryptosporidium baileyi*, is the species most isolated from broilers, which is responsible for respiratory disorders and bursa of Fabricius inflammations, while the second, *Cryptosporidium meleagridis*, is the avian zoonotic species that mainly causes intestinal disease in birds (Feng and Xiao 2011), *C.* *galli*, the third species, is located at the proventriculus and unusually, *C.* *parvum*, a mammalian zoonotic species, has been detected in broilers in some studies (Al-Ghezey and Al-Zubaidi 2020). In broiler chickens, data are lacking on *Giardia* infections, though a few studies that have been conducted have revealed the presence of *G.* *duodenalis* (Cao et al. 2020).

In Algeria, in ruminants, despite the availability of some data on* Cryptosporidium* (Baroudi et al. 2017; Ouakli et al. 2018; Saidi et al. 2020; Benattia et al. 2024) and on *Giardia* infections (Baroudi et al. 2017), there are only a few studies that have been undertaken in chickens (Baroudi et al. 2013; Guechtouli et al. 2022) while there is no information on *Giardia* in birds. The objective of this study is to assess the prevalence of *Cryptosporidium* and *Giardia* in broiler chickens and calves and to determine some of the associated risk factors.

## MATERIAL AND METHODS

### Study area and sampling

The survey was undertaken in 2023 in Cheraga province, in the western part of Algiers (Latitude: 36.7538° N, Longitude: 3.0588° E). Cheraga is considered an agricultural area. The study was carried out in the rural part where poultry and cattle farms exist. In total, 15 cattle and 10 broiler chicken farms were visited, where the cattle ranged between 10 and 20 and the chickens ranged between 2 000 to 3 000 subjects at each farm. The cattle farms and chicken buildings were independent on each other. The cattle included in the study lived in barns where the hygienic conditions are acceptable. The removal of manure and faeces was regularly performed. In the chicken farming, the droppings were eliminated every three days, when they accumulated.

A total of 100 faecal samples were collected from 10 different places for each broiler chicken building (10) (*n* = 10 × 10), the birds were aged from 17 to 52 days. The samples from the calves (*n* = 100) were randomly taken from cattle aged 10 to 150 days across 15 farms.

### Experimental design

For the *Giardia* and *Cryptosporidium* spp. detection, we used the Ritchie technique simplified by Allen and Ridley as previously described (Bennadji et al. 2022), and modified Ziehl-Neelsen staining (Henriksen and Pohlenz 1981). The avian (*n* = 100) and bovine samples (*n* = 100) were subjected to optical microscopy. Among the collected samples, sixty (30 avian and 30 bovine samples) were tested with a Giardia^®^ rapid immunochromatographic kit speed to confirm the microscopic results. This is a rapid test that allowed for the detection of *Giardia duodenalis* cyst antigens with a sensitivity of 95.6% and a specificity of 100%.

Faecal samples were collected from the rectum of the cattle by stimulating the anal orifice. Regarding sampling within the poultry buildings, the faecal samples were collected around the feeders and drinkers. All the samples were placed in sterile transparent containers and coded, reporting the necessary information for each sample, such as the sex and age of the animals.

**Figure 1:**
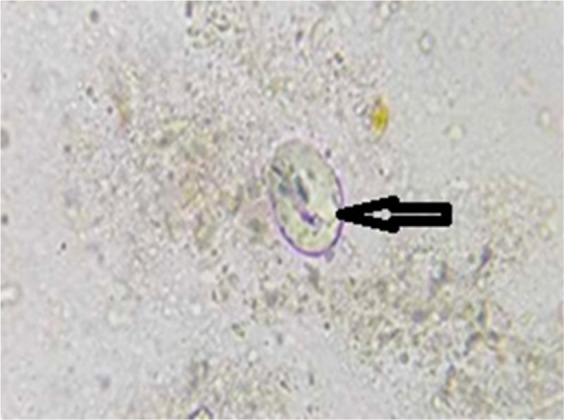
Cyst of *Giardia* spp. (arrow) observed under light microscope at × 40 magnification after concentration with the formol-ether method

**Figure 2:**
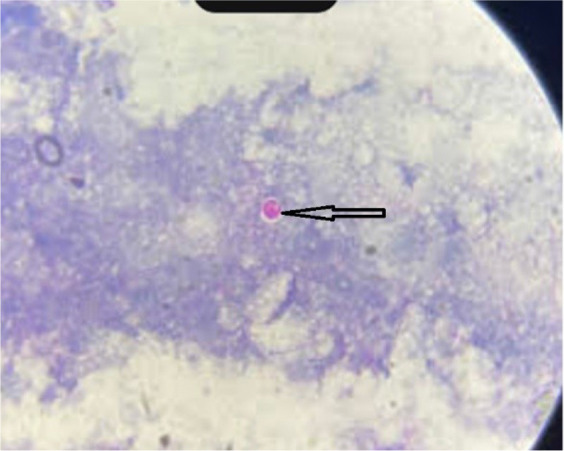
*Cryptosporidium* oocyst (arrow) seen under a light microscope at 100 Â magnification (immersion), the stool smears were stained with the Ziehl-Neelsen modified method

*Giardia* cysts were detected under a light microscope with × 40 magnification (Figure 1). To verify the presence of *Cryptosporidium* spp. oocysts, observation of the stained slides was performed at × 40 magnification and then at × 100 magnification after the addition of immersion oil (Figure 2).

Regarding the statistical analysis, the chi-square test with a confidence range of 95% was performed to compare the prevalence in relation to the age, sex and species. The differences were considered statistically significant when *P* ≤ 0.05 with a confidence range of 95%.

### Ethical statement

All the animal studies were conducted with the utmost regard for animal welfare, and all animal rights issues were appropriately observed. No animal suffered during the the work. All the experiments were carried out according to the guidelines of the Institutional Animal Care Committee of the Algerian Higher Education and Scientific Research (Agreement No. 45/DGLPAG/DVA.SDA. 14).

## RESULTS AND DISCUSSION

### Microscopic analysis

The microscopic examinations using the simplified Ritchie technique for *Giardia* and the Ziehl-Neelsen staining for *Cryptosporidium* spp. revealed the presence of cysts and oocysts of both parasites (Figure 1 and Figure 2, respectively).

### Broiler chickens

#### OVERALL PREVALENCE

In the broiler chickens, the data showed that 60% of the birds were infected by *Cryptosporidium* spp., while *Giardia* was not found in the faeces (0%) (Table 1).

**Table 1:** Prevalence of *Cryptosporidium* spp. and *Giardia* according to the age of the broiler chickens

Age (days)	Samples (*n*)	*Cryptosporidium* spp.+ (*n*)	*Cryptosporidium* spp. (%)	Samples (*n*)	*Giardia*+ (*n*)	*Giardia* (%)
17	8	6	75	8	0	0
20	12	2	16.66	12	0	0
24	12	10	83.33	12	0	0
27	8	8	100	8	0	0
28	12	10	83.33	12	0	0
30	48	24	50	48	0	0
						
Total	100	60	60	100	0	0

χ² = 60, *P* < 0.000 1

Regarding the *Cryptosporidium* spp. prevalence (60%), our results align with those found in Algeria (Guechtouli et al. 2022) with the rates of 55% and 50%. However, the prevalence is higher than those reported in Nigeria (19%) (Bamaiyi et al. 2016), in Algeria (34%) (Baroudi et al. 2013), and in Tunisia (4.5%) (Soltane et al. 2007). A lower prevalence was also revealed in a recent work in China (2.43%) (Cao et al. 2020). The difference with other studies worldwide can be explained by several factors: the geographical location and the number of animals and farms monitored (Bennadji et al. 2022), which differ considerably from one study to another.

The statistical analysis indicates a significant difference in the infection rates, with *Cryptosporidium* showing a 60% positivity rate (χ² = 60, *P* < 0.000 1), while *Giardia* has a 0% positivity rate.

This suggests that *Cryptosporidium* is much more prevalent than *Giardia* in broiler chickens. The findings highlight that *Cryptosporidium* is a common parasite in this population, whereas *Giardia* is not present. It is well known that once present in a few animals, *Cryptosporidium* infections quickly become widespread. *Cryptosporidium* oocysts are directly infective with a high capacity for resistance in the environment (Benattia et al. 2024). Moreover, the oocysts of these *Coccidia* are known for their extremely frequent self-infestation capability, which justifies the high rate (Saidi et al. 2024).

#### PREVALENCE ACCORDING TO AGE

Regarding the *Giardia* infestation, no animals of any age excreted this parasite. In contrast, for *Cryptosporidium* spp. (Table 1), all animals aged 27 days excreted the parasite (100%). Animals aged 24 days and 27 days had the same rate (83.33%), followed by the animals aged 17 days (75%) and those aged 30 days (50%). The lowest rate (16.66%) was identified in animals aged 20 days.

Relating to *Cryptosporidium* spp. in animals aged between 17–30 days, these results join those found by Goodwin and Brown (1988) in chickens of 17–52 days, in the USA. Furthermore, *Cryptosporidium* spp. infections are generally found in animals aged less than 11 weeks and not less than 3 weeks, especially during the period of rapid growth (16–30 days) (de Graaf et al. 1999; Baroudi et al. 2013).

Moreover, the statistical analysis (χ² = 18.14, *P* = 0.001 1) showed a statistically significant difference between the *Cryptosporidium* spp. excretion rates among the different age groups. This indicates that age has a significant effect on the prevalence of the *Cryptosporidium* spp. infection. This relationship can be explained by the poor hygienic conditions at the hatchery as a large percentage of chicks were affected by omphalitis. These may have favoured the infestation through a debilitating action on the immune system (Bennadji et al. 2022). This rate remains significantly lower than those recorded between 10 and 50 days of age due to the resistance of animals, which are likely have antibody levels against *Cryptosporidium*, and the acquisition of immunity (the “age” and “immunity” factors are linked and intervene concomitantly) (Guechtouli et al. 2022).

### Cattle

#### OVERALL PREVALENCE

Regarding the infections in cattle, a rate of 56% of *Cryptosporidium* spp. and 8% of *G. duodenalis,* were observed. These results are significant and reflect the parasitic prevalence in cattle breeding. Other studies have shown that cattle have a higher *G. duodenalis* infestation rate (Xiao et al. 1993; Olson et al. 1997; Kadukova et al. 2024).

The high rate of *Cryptosporidium* is in accordance with previous data. In act, the prevalence in cattle varied between 13% and 93% with an overall average of 25.5% depending on the region (Buchanan et al. 2024). As for *G.* *duodenalis*, although the rate found is low, it remains significant; previous data have reported a worldwide prevalence ranging between 5% and 35% in cattle (Mateusa et al. 2023). The difference in the infection rates between *Cryptosporidium* and *G. duodenalis* could be explained by biological factors: *Cryptosporidium* oocysts are more resistant in the environment than *Giardia* cysts, facilitating its potential transmission (Wang et al. 2023). In addition, *Cryptosporidium parvum* has a shorter life cycle and can multiply more rapidly in the host than *G. duodenalis*. These findings underscore the importance of the ongoing surveillance and the implementation of preventive measures in cattle farming. Indeed, high animal densities and inadequate sanitary conditions contribute to the risk of infection (Guechtouli et al. 2022).

The statistical analysis revealed that *Cryptospo-ridium* spp. represents a significantly higher risk factor for infection in the studied cattle compared to *G. duodenalis*. The chi-square test (χ² = 36, *P* < 0.000 01) and the odds ratio (OR ≈ 14.64) indicate that cattle are about 14.64 times more likely to be infected with *Cryptosporidium* oocysts than with *Giardia* cysts. This substantial disparity suggests the existence of specific risk factors favouring *Cryptosporidium* infections, such as its potentially increased environmental resistance, more efficient transmission modes, greater host susceptibility, or farming practices that might facilitate its spread (Saidi et al. 2020).

Regarding the prevalence of *Cryptosporidium* spp., previous studies performed in Algeria indicated low rates (Ouchene et al. 2012; Baroudi et al. 2017). Our results are in agreement with those reported by Quilez et al. (1996), Ouchene et al. (2014), and Thomson et al. (2017) with rates of 63%, 50.8%, respectively. In the UK, the authors mentioned intervals of 28–80% (Thomson et al. 2017). Relating to *Giardia*, the prevalence is lower than those estimated previously (Quilez et al. 1996; Ouchene et al. 2012) with rates of 11.7%, and 15.09%, respectively.

Our findings present a co-infection in four animals, corresponding to a rate of 4% (4/100). This result aligns with a co-infection value of 4.8% in calves in a recent study reported in Iran (Mirhashemi et al. 2015). Contrary to earlier reports suggesting competition between *Giardia duodenalis* and *Cryptosporidium* spp. in calves (Hamnes et al. 2006), recent research provides a more nuanced perspective on this parasitic interaction. It revealed the complexity of interactions between these parasites. Rather than simple competition, there is potential evidence of synergy, so a *Giardia* infection might facilitate *Cryptosporidium* colonisation by modifying the intestinal barrier. Moreover, a co-infection could worsen the symptom severity and extend the infection duration (Ryan et al. 2014). This can engender a public health issue, particularly among children, thus the structural damage inflicted by both parasites to the epithelial cells in the large and small intestines could severely impair the children’s gut health, including the ability to absorb nutrients, resulting in stunted growth, diminished neurocognitive development, and other long-term effects (Prabakaran et al. 2023)

This co-infection can be due to various factors. In fact, these parasites share similar environmental conditions and comparable transmission modes, thriving in humid environments and contaminating water sources (Thomson et al. 2019). Additionally, young animals, mainly calves, are more susceptible to multiple infections because their immune system is immature (Geurden et al. 2010). The presence of these co-infections underscores the importance of comprehensive diagnostics and adapted therapeutic approaches, as certain treatments may be efficacious against one parasite, but not the other. These observations highlight the necessity for continued research into the complex interactions between these parasites to enhance our understanding and management of parasitic infections in animals.

#### PREVALENCE ACCORDING TO SEX

Regarding the infestation according to sex, the results showed a rate of 0% in females for *Giardia* (0%) (Table 2).

**Table 2:** Prevalence of* Giardia* according to sex in cattle

Sex	Samples (*n*)	*Giardia duodenalis*+ (*n*)	*Giardia duodenalis* (%)
Female	60	0	0
Male	40	8	20
			χ² = 22.22 *P* < 0.000 1

However, the absence of *Giardia* did not agree with most of the published findings (Thompson 2004; Feng and Xiao 2011). This discrepancy underscores the complex nature of host-parasite interactions and highlights the potential influence of factors such as environmental conditions and host-parasite specificity.

In fact, patterns of sex-biased parasitism can vary not only among host taxa, but also among populations of the same species, reflecting the complex interaction of genetic, environmental, and ecological factors (Schalk and Forbes 1997). These results underline the need for further investigation, including larger-scale and longitudinal studies, to reveal the mechanisms driving these sex-specific infection patterns and to better understand their implications for parasite epidemiology and host health.

Also, a sex dependent variation in the parasitic infestation was observed in our study, with males showing a higher susceptibility to *Giardia* spp. Specifically, the males revealed a prevalence of 20% for *Giardia* spp. These findings suggest a potential male predisposition in parasitic infections, for *Giardia* spp.

Our observations align with recent studies that have reported sex-based differences in protozoan infections, while a predisposition towards higher *Giardia* spp. infection rates in males has been reported by other authors (Koehler et al. 2014).

However, the relationship between the host sex and parasitic infection is complex and can vary across different host species and environmental contexts. Contrasting our findings, a higher prevalence of *Giardia* spp. in female ruminants was reported by Squire et al. (2017) in Ghana. These disparities underscore the multifactorial nature of parasitic infections and the need for context-specific investigations.

The statistical analysis of the *Giardia* infestation revealed a significant difference in infestation rates between the sexes, as indicated by the chi-square test (χ² = 22.22, *P* < 0.000 1). The results demonstrate a significant association between the sex and *Giardia* infestation, with males exhibiting a higher predisposition to infestation compared to females. These findings suggest that sex is a significant risk factor for *Giardia* infestation.

#### PREVALENCE ACCORDING TO AGE

Regarding the infestation rate according to the age of the cattle, the highest value was found in the animals aged 10–26 days with a rate of 80% for *Cryptosporidium* spp., while *Giardia* was not found (0%). This was followed by animals aged 30–60 days with a prevalence of 64% for *Cryptosporidium* spp. and 14.28% for *Giardia*. Finally, for cattle aged 75–120 days, the prevalence for *Cryptosporidium* spp. reached 17%, while *Giardia* was not isolated (0%) (Table 3). Garcia et al. (2020) noticed that calves are particularly susceptible to infection, with rates as high as 100% in some herds. The results obtained showed that infestations by *Cryptosporidium* spp. occur mainly at an early age, while those due to *Giardia* occur later. These results are consistent with a previous study (Del Coco et al. 2008).

**Table 3:** Prevalence of *Cryptosporidium* spp. and *Giardia* according to the age of the cattle

Age (days)	*n*	*Cryptosporidium* spp.+ (*n*)	*Cryptosporidium* spp. (%)	*Giardia duodenalis*+ (*n*)	*Giardia duodenalis* (%)
10–26	20	16	80	0	0
30–60	56	36	64	8	14.28
75–150	24	4	17	0	0
			χ² = 138.43 *P* < 0.000 1		χ² = 15 *P* = 0.000 5

Our data showed that in 84% of calves infested by *Cryptosporidium* spp., the animals were less than one month old, which is in agreement with the findings reported previously (Thomson et al. 2017) which cited that this infestation occurs in subjects aged between 4 days and 1 month. In the same context, calves are considered one of the main reservoirs of zoonotic *Cryptosporidium* spp. and contact with them can pose a possible risk of infection, especially for farm workers, animal technicians, veterinarians, and veterinary medicine students (Kadukova et al. 2024).

Regarding the infestation by *Giardia* spp., the results obtained are consistent with those of other investigators who have also observed that the parasite excretion is, at its maximum in calves aged between 1 and 2 months. Other studies have reported a maximum excretion between 1.5 and 4 months in calves (Quilez et al. 1996). It is well documented that young animals, due to their less developed (immature) immune systems, can be more susceptible to parasitic infestations, in agreement with previous reports (Benattia et al. 2024).

In conjunction with a conventional microscopic examination, we used a commercially available immunochromatographic assay, Speed *Giardia*®, for enhanced parasite detection. When applied to the avian faecal samples that yielded negative results through the microscopic analysis, the immunoassay demonstrated concordant findings. All the samples (*n* = 30) remained negative, with the rapid immunochromatographic method failing to detect any parasitic antigens. This corroboration between the microscopy and immunoassay results in avian samples suggests either the absence of *Giardia* or parasite loads below the detection threshold of both methods. Conversely, the application of the commercial immunoassay kit to bovine samples (*n* = 30) that were initially deemed negative via microscopic examination revealed the presence of the parasitic antigen in six samples, indicating the higher sensitivity of the immunological method compared to conventional microscopy. This result may be due to the fact that the commercial kit is highly sensitive and consequently able to detect subclinical infections.

The statistical analysis showed that the differences in the *Cryptosporidium* spp*.* infestation rates among the age groups were statistically significant (χ² = 138.43, *P* < 0.000 1), the animal’s age has a significant impact on the prevalence of the *Cryp-tosporidium* spp. infection. Regarding the *Giardia* infestation, the statistical analysis revealed that the differences in the *Giardia* infestation rates among the age groups were also statistically significant (χ² = 15, *P* = 0.000 5), which confirms the influence of this parameter on the *Giardia* infestation.

The statistical analysis revealed a significant difference (χ² = 24, *P* < 0.000 01) between the *Giardia* prevalence detected by microscopy (8%) and the Speed Giardia^®^ test (20%) in the calves. This suggests that the Speed Giardia^®^ test has a higher sensitivity compared to the microscopy for the *Giardia* detection in calves.

Regarding the broiler chickens, since *Giardia* was not detected by microscopy (0% prevalence), the chi-square test cannot be performed to compare the two methods. The Speed Giardia^®^ test did not identify any positive samples in chickens, which is consistent with the microscopy findings. The statistical analysis revealed that the Speed Giardia^®^ test is significantly more sensitive than the microscopy for the *Giardia* detection in calves, while both methods consistently showed a lack of *Giardia* infestation in broiler chickens.

This current study indicated that *Cryptosporidium* spp. and *Giardia* are common in broiler chickens and calves. *Cryptosporidium* spp. was identified with a high prevalence in broiler chickens, primarily affecting young chicks. In contrast, *Giardia* was not detected in this species. A similar prevalence of *Cryptosporidium* was detected in calves, whereas *Giardia* was significantly more prevalent in this species. The sex and age are considered risk factors for *Giardia* infestations in cattle. The age is a significant risk factor for both parasites, with opposing effects: the risk of *Cryptosporidium* decreased with age, while that of *Giardia* increases. Sex is not a significant risk factor for *Cryptosporidium* in calves, but males have a significantly higher risk of *Giardia* infection. The use of a rapid *Giardia duodenalis* detection test allowed its detection in cattle samples, indicating that it is interesting to combine a rapid test with microscopy for confirming infestations in animals. The Speed Giardia^®^ test appears to be more sensitive than microscopy. It is important to emphasise that the epidemiology of these parasites in livestock, particularly for *Giardia*, remains insufficiently studied. Other risk factors such as bedding, hygiene, contact with the mother, and diet should be explored to implement the necessary preventive measures to eliminate these pathogens in livestock farms in Algeria.
